# Dyspepsia Cure

**Published:** 1874-10

**Authors:** 


### DYSPEPSIA CURE.

TAKE a grindstone, a table knife and a good dinner. Eating a plenty of good things seldom fails to cure the worst cases of dyspepsia, if properly done. Many have starved themselves into more aggravated forms of the disease, under the delusive impression, that as the malady is caused by eating too much, the best cure is to eat nothing, or something next door to it. All natural strength comes from something good to eat, and can come from no other source.

Dyspepsia is weakness of the stomach; it has not the strength to digest the food properly. If then, you do not give the dyspeptic stomach enough to eat, it grows weaker, and the disease becomes worse. It therefore follows, that there can be but one natural and reasonably speedy cure of dyspepsia, and that is to eat a plenty of good things, which means nourishing food; food which is made to taste well; "taste good" to the patient. It was not intended to advise the dyspeptic to eat a grindstone or swallow the table knife, nor should the knife be used to slice off the grindstone, as a loaf of bread for table use; but the grindstone is to sharpen the knife, and the knife is to cut up all solid food, in pea sized pieces; and if this food is the best, is taken not oftener than two or three times a day, never an atom between meals, a dyspeptic will begin to get well in a week.

The sharp knife cuts the food; thoroughly dividing all the strings of meat and vegetables, and the hard lumps of other food. If this is well done, and the food is leisurely chewed, the first result is, we enjoy more of its taste, as we get the juices out of every particle; and next, it is more perfectly and more speedily digested after it gets into the stomach, just as a large lump of ice, cut into pea sized pieces and thrown into half a glass of water dissolves more rapidly than if undivided; for the ice in the glass, like the bits of food in the stomach, is dissolved from without inwards, a greater surface being exposed to the water.

There is no virtue in a person punishing himself by eating what is disagreeable to him ; it is a positive injury, for other things being equal, that is more easily digested, which is eaten with a relish, than that which is distasteful. Sometimes an article of food disagrees with the stomach which most agrees with the taste ; but it is best for the stomach for all that, because instinct craves it; the error is in eating too much of it at a time; diminish the amount at each meal, until it is ascertained how much can be taken without any discomfort whatever ; continue that amount for a few days, and then increase it very gradually. Fruits are best for dyspeptics ; next to that fresh meats ; and vegetables third.

